# Chronic Stress Leads to Time-Dependent Bone Loss Through HPA Axis Dysregulation and GR Nuclear Translocation Disorder

**DOI:** 10.3390/ijms27031449

**Published:** 2026-01-31

**Authors:** Yupeng Yan, Jiaxin Li, Zhengmin Lu, Zhiguo Zhang, Gaimei Hao, Yukun Zhao, Haixia Liu, Yanjun Liu, Xiangxin Bao, Mengya Duan, Yubo Li

**Affiliations:** Institute of Basic Theory for Chinese Medicine, China Academy of Chinese Medical Sciences, Beijing 100700, China; albertyan727@gmail.com (Y.Y.); lijiaxin121024@163.com (J.L.); imnotminmin927@gmail.com (Z.L.); zzgtcm@163.com (Z.Z.); haogaimei@163.com (G.H.); zhyk_87@163.com (Y.Z.); 18810485141@163.com (H.L.); liuyj@ibtcm.ac.cn (Y.L.); baoxiangxin0113@163.com (X.B.); 17596520819@163.com (M.D.)

**Keywords:** chronic stress, bone loss, depression, HPA axis, glucocorticoid receptor, glucocorticoid resistance

## Abstract

Chronic stress and sustained hypothalamic–pituitary–adrenal (HPA) axis activation are major contributors to metabolic bone diseases, including osteoporosis. However, the precise molecular mechanisms by which chronic stress-induced HPA axis dysregulation drives bone deterioration remain unclear. A Chronic Unpredictable Mild Stress (CUMS) model was established in male rats to simulate prolonged stress exposure. Animals were randomly allocated into three groups: control, 10-week CUMS, and 20-week CUMS (*n* = 10/group). Model validity was confirmed via behavioral assessments. Bone mineral density (BMD) and trabecular microarchitecture were quantified using micro-computed tomography (micro-CT). Serum corticosterone (CORT) levels, HPA axis negative feedback function, and the expression of pro-inflammatory cytokines (IL-1β, TNF-α) in HPA-regulatory brain regions (hippocampus, prefrontal cortex, hypothalamus) were assessed. Critically, glucocorticoid receptor (GR) expression and nuclear translocation in these brain regions and bone tissue were examined by immunofluorescence and Western blot analysis. CUMS exposure induced progressive, time-dependent bone loss, with the 20-week group exhibiting significantly greater reductions in BMD and trabecular quality compared to the 10-week and control groups. While the HPA axis showed initial hyperactivation, the 20-week group displayed adrenal exhaustion (reduced serum CORT) alongside elevated ACTH, indicating feedback failure. Mechanistically, stress significantly impaired GR nuclear translocation in both brain and bone tissues, coinciding with the upregulation of FKBP5 and pro-inflammatory cytokines. Notably, despite low systemic CORT at late stages, skeletal 11β-HSD1 expression was significantly upregulated, creating a local microenvironment of glucocorticoid toxicity that aggravated osteoblast apoptosis. Our findings demonstrate that chronic stress induces progressive, time-dependent bone loss through a cascade of HPA axis dysregulation and impaired GR signaling. The FKBP5-mediated impairment of GR nuclear translocation in both central and peripheral tissues fosters glucocorticoid resistance, perpetuating hypercortisolemia and a pro-inflammatory milieu that directly accelerates osteoblast apoptosis and bone deterioration. These findings identify the HPA-GR axis as a critical pathway linking chronic stress to osteoporosis and suggest that restoring GR signaling offers a potential therapeutic strategy.

## 1. Introduction

Stress is a recognized precursor to numerous diseases. While acute stress triggers adaptive responses, chronic stress detrimentally impacts health by overburdening adaptive systems. Clinically, chronic stress is associated with multiple systemic pathologies [1,2,3,4,5] and is implicated in skeletal deterioration. Osteoporosis and depression frequently co-occur, particularly in aging populations [6], suggesting potential shared mechanisms involving stress. Given that osteoporosis management focuses on mitigating bone loss [7], identifying risk factors such as chronic stress—clinically linked to reduced bone mineral density [8,9,10]—is essential and warrants mechanistic investigation in animal models.

Chronic stress primarily exerts its effects on target organs via neuroendocrine pathways, predominantly the hypothalamic–pituitary–adrenal (HPA) axis. Under physiological conditions, HPA-mediated cortisol release regulates osteoblast function and modulates inflammation. However, sustained stress can impair HPA axis negative feedback, leading to chronic hypercortisolemia. This pathological state is associated with systemic damage and potentially reduced glucocorticoid receptor (GR) nuclear translocation. Such signaling dysfunction promotes glucocorticoid resistance [11] and chronic inflammation [12], thereby compromising bone homeostasis. Since effective glucocorticoid signaling hinges on GR nuclear translocation, its disruption presents a plausible mechanism for stress-induced bone pathology. Accordingly, this study investigates whether chronic stress drives bone loss through HPA axis dysregulation and subsequent impairment of GR nuclear translocation.

## 2. Results

### 2.1. Effects of Chronic Stress on Rat Behavior

**Figure 1:**
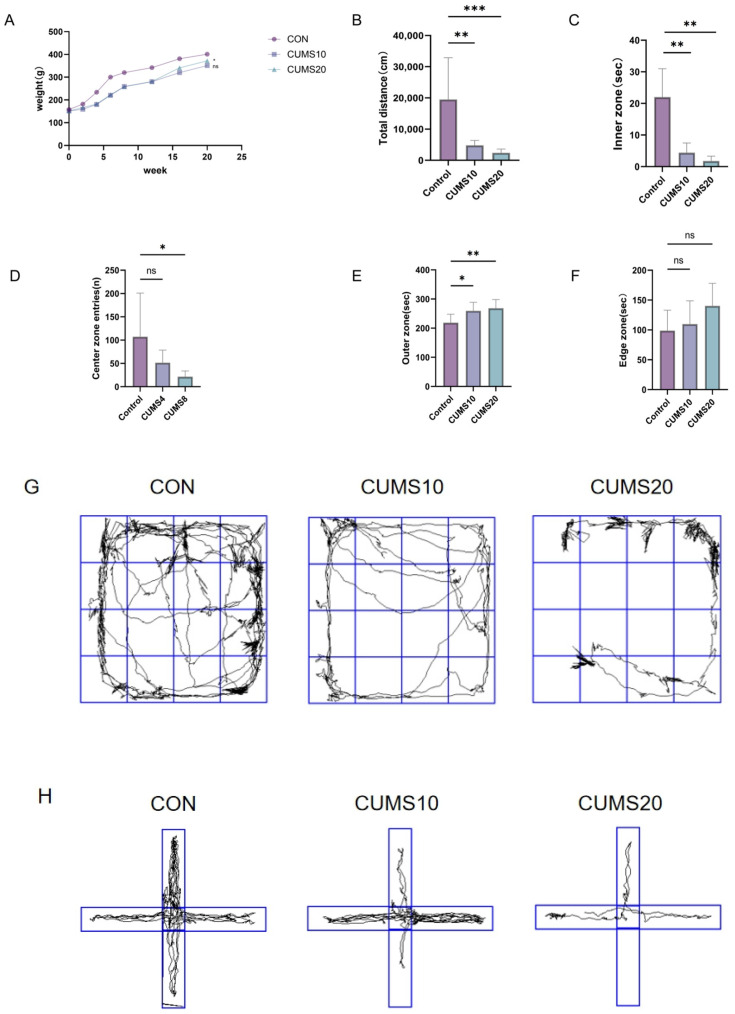

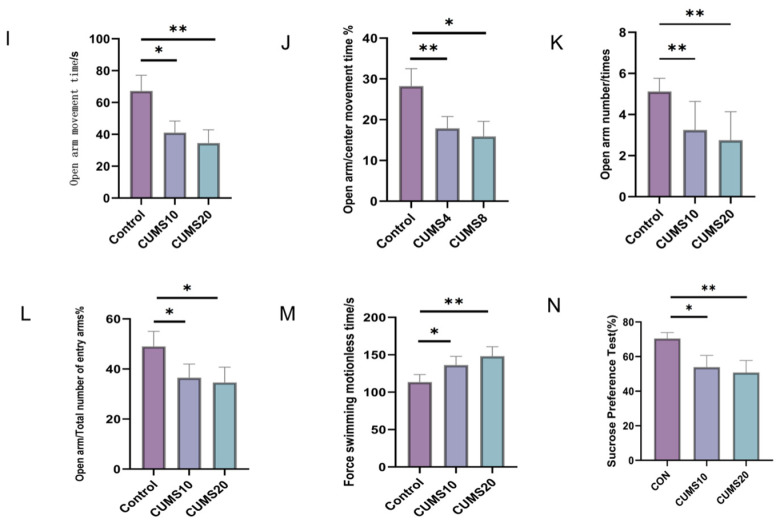
Depression Levels Intensify with the Duration of Chronic Stress. (**A**) Body weight increase curves for each group (*n* = 8, * *p* < 0.05, ns > 0.05); (**B**–**G**) Open Field Test (OFT): Total distance, center activity time, number of crossings, time spent in the periphery, time spent in the corners, and tracks of different groups (*n* = 8, ** *p* < 0.01, * *p* < 0.05, *** *p* < 0.001, ns > 0.05, ns: no significant difference); (**H**–**L**) Elevated Plus-Maze Test (EMP): Tracks, open arm movement time ratio, open arm/closed arm time ratio, number of entries into the open arms, and the ratio of open arm entries to total entries (*n* = 8, ** *p* < 0.01, * *p* < 0.05); (**M**) Forced Swim Test (FST): Immobility time during the 5 min test (*n* = 8, ** *p* < 0.01, * *p* < 0.05); (**N**) Sucrose Preference Test (SPT): Preference for sucrose solution (*n* = 8, ** *p* < 0.01, * *p* < 0.05).

No significant differences in body weight were observed between groups, although a non-significant trend towards lower weight was noted in CUMS-exposed rats compared to controls (Figure 1A).

Behavioral assessments consistently revealed stress-induced alterations. In the Open Field Test (OFT; Figure 1B–F), both CUMS groups (10 and 20 weeks) demonstrated significantly reduced exploratory behavior (total distance, center time, crossings; *p* < 0.05) and increased peripheral dwelling time (*p* < 0.05) versus controls, indicative of heightened anxiety and decreased locomotion. Corner time did not differ significantly (*p* > 0.05).

Similarly, the Elevated Plus Maze (EPM; Figure 1H–L) showed that CUMS exposure significantly decreased open arm exploration (distance and time; *p* < 0.05) while increasing closed arm duration (*p* < 0.05) compared to controls. Notably, the CUMS 20-week group exhibited further significant reductions in the ratio of open arm entries and the open/closed arm time ratio (*p* < 0.01 vs. controls), suggesting exacerbated anxiety-like behavior with prolonged stress.

In the Forced Swim Test (FST; Figure 1M), immobility time was significantly elevated specifically in the CUMS 20-week group compared to controls (*p* < 0.01), indicating increased behavioral despair.

Finally, the Sucrose Preference Test (SPT; Figure 1N) confirmed stress-induced anhedonia, as CUMS-exposed rats displayed a significantly reduced preference for sucrose solution compared to controls.

### 2.2. Effects of Chronic Stress on HPA Axis-Related Indicators in Rats

**Figure 2:**
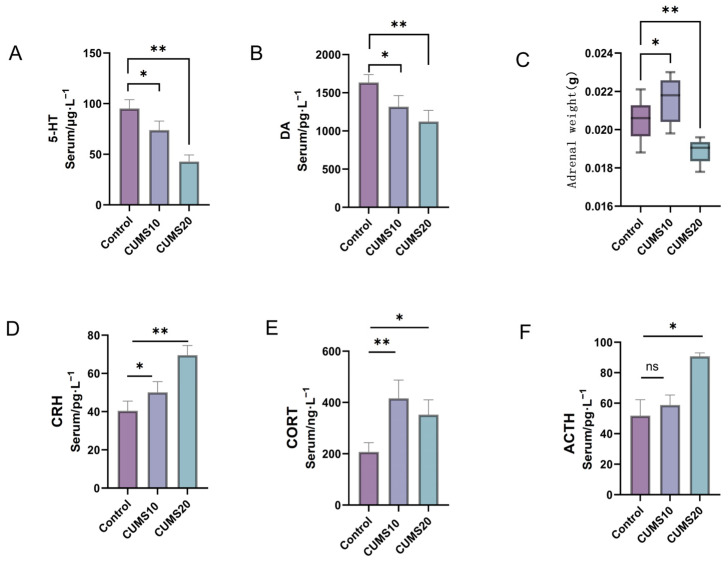

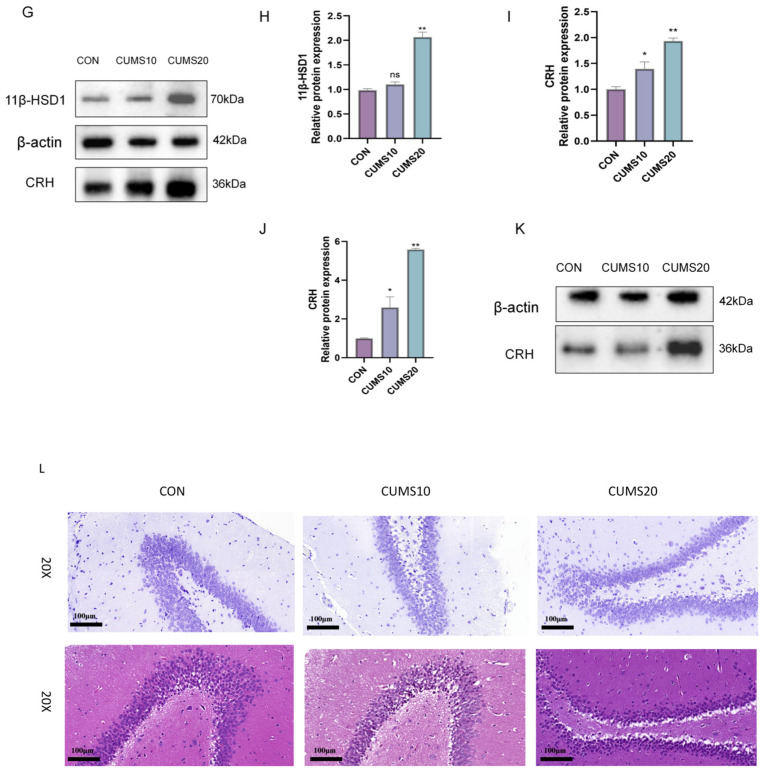

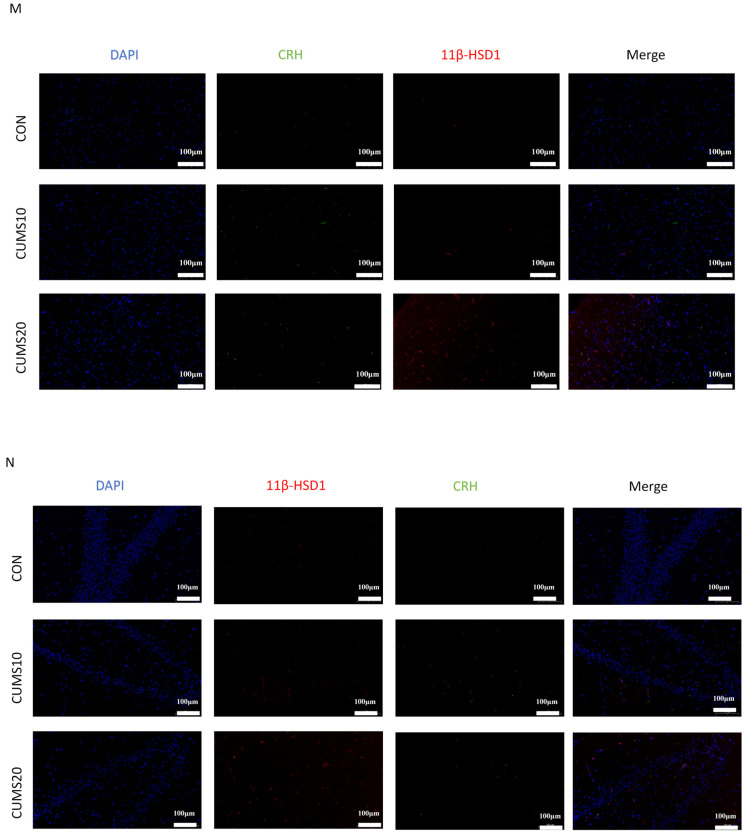
Evaluation of Neurotransmitter Reduction and Neuronal Damage in the Hippocampus of Rats with HPA Axis Dysfunction Induced by Chronic Stress at Different Time Points. (**A**,**B**) The relative levels of serotonin (5-HT) and dopamine (DA) in the blood serum of rats across different groups (*n* = 6, *p* * < 0.05, *p* ** < 0.01); CUMS: Chronic Unpredictable Mild Stress; (**C**) Adrenal gland weight of rats (*n* = 6, *p* * < 0.05, *p* ** < 0.01); (**D**–**F**) The relative levels of CRH (Corticotropin-releasing hormone), CORT (Corticosterone), and ACTH (adrenocorticotropic hormone) in the blood serum of rats across different groups (*n* = 6, *p* * < 0.05, *p* ** < 0.01, ns > 0.05); (**G**–**I**) Western blot analysis was performed to detect the expression of CRH and 11β-HSD1 proteins in the hippocampal tissue of rats across different groups (*n* = 3, *p* * < 0.05, *p* ** < 0.01, ns > 0.05, ns: no significant difference); (**J**,**K**) Western blot analysis was performed to detect the expression of CRH protein in the hypothalamic tissue of rats across different groups (*n* = 3, *p* * < 0.05, *p* ** < 0.01); (**L**) Hematoxylin and eosin (HE) staining of the hippocampus (20×); (**M**) Immunofluorescence double staining for CRH/11β-HSD1 in the prefrontal cortex of rats; (**N**) Immunofluorescence double staining for CRH/11β-HSD1 in the hippocampus of rats.

ELISA revealed significantly reduced levels of dopamine and serotonin in CUMS-exposed rats compared to controls (Figure 2A,B; *p* < 0.05), with the most pronounced decrease observed in the CUMS 20-week group (*p* < 0.01 vs. controls).

Analysis of the HPA axis indicated progressive dysregulation. Compared to controls, serum CRH and CORT levels were significantly elevated at 10 weeks (Figure 2D,F; *p* < 0.05), accompanied by increased adrenal weight (Figure 2C; *p* < 0.05), suggesting initial HPA activation. Serum ACTH levels, however, were not significantly different from controls at this time point (Figure 2E; *p* > 0.05). By 20 weeks, serum CRH and ACTH levels were significantly elevated compared to both control and 10-week groups (Figure 2D,E; *p* < 0.05), yet serum CORT levels were significantly lower than at 10 weeks (Figure 2F), coinciding with a marked reduction in adrenal weight suggestive of atrophy (Figure 2C; *p* < 0.01). This temporal dissociation between pituitary (ACTH) and adrenal (CORT) output at 20 weeks suggests impaired HPA axis negative feedback and potential adrenal exhaustion.

Western blot analysis demonstrated time-dependent upregulation of key proteins. In the hippocampus (Figure 2G–I), At 10 weeks, CRH expression significantly increased (*p* < 0.05 vs. control), whereas 11β-HSD1 showed only a mild, non-significant trend (*p* > 0.05). Subsequently, CRH levels were markedly further elevated at 20 weeks (*p* < 0.01 vs. 10 weeks and control). Hypothalamic CRH protein expression also increased following CUMS (Figure 2J,K). Immunofluorescence (Figure 2M,N) corroborated these findings, showing a significant increase in 11β-HSD1 and CRH immunopositivity in the prefrontal cortex and hippocampus at 10 weeks, with further enhancement at 20 weeks.

HE staining (Figure 2L) showed evident hippocampal atrophy specifically in the CUMS 20-week group compared to controls and the CUMS-10 week group (which appeared morphologically similar to controls). However, Nissl staining indicated neuronal damage in both CUMS groups, characterized by irregular neuronal arrangement, reduced cell density, and vacuolization indicative of necrosis.

### 2.3. Effects of Long-Term Chronic Stress on GR Nuclear Translocation in Rats

**Figure 3:**
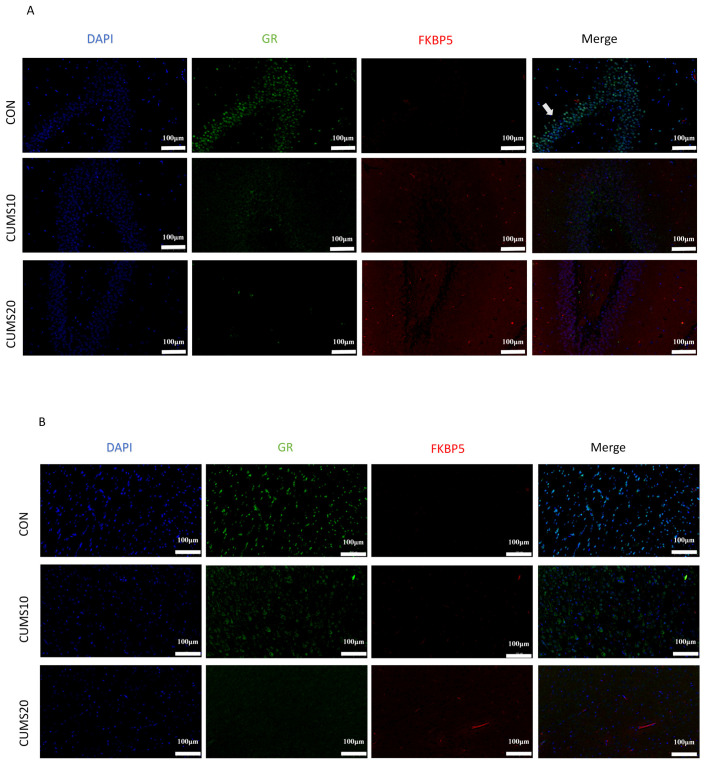

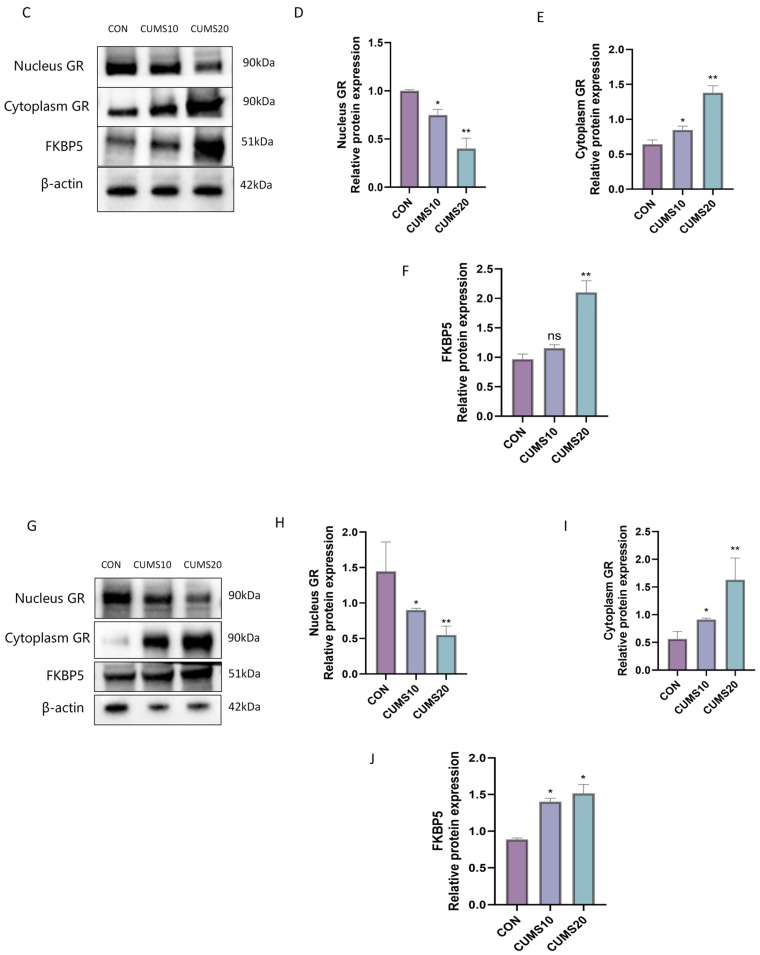
Time-Dependent Reduction in GR Nuclear Translocation Induced by CUMS in Rats. (**A**,**B**) Immunofluorescence double staining for GR/FKBP5 in the hippocampus and prefrontal cortex of rats, The arrow indicates the GR/FKBP5 fluorescence co-location; (**C**–**F**) Western blot analysis was performed to detect the expression of nuclear GR, cytoplasmic GR, and FKBP5 (FK506-binding protein 5) proteins in hippocampal tissue across different groups (*n* = 3, *p* * < 0.05, *p* ** < 0.01, ns > 0.05, ns: no significant difference); (**G**–**J**) Western blot analysis was performed to detect the expression of nuclear GR, cytoplasmic GR, and FKBP5 proteins in hypothalamic tissue across different groups (*n* = 3, *p* * < 0.05, *p* ** < 0.01).

Western blot analysis assessed GR nuclear translocation in the hippocampus and hypothalamus of rats (Figure 3C–E). After 10 weeks of CUMS exposure, GR expression in the hippocampal nucleus was significantly reduced compared to normal rats (*p* < 0.05), while cytoplasmic GR expression increased (*p* < 0.01). After 20 weeks of CUMS exposure, nuclear GR expression in the hippocampus was significantly lower than in the CUMS 10-week group. Furthermore, FKBP5 expression (Figure 3F) was significantly increased after 20 weeks of CUMS exposure (*p* < 0.01), indicating that chronic stress disrupts GR nuclear translocation.

Immunofluorescence (IF) double staining for GR/FKBP5 (Figure 3A,B) provided further insight into the hippocampus and prefrontal cortex. In the control group, GR expression was confined to the nucleus and around the cytoplasm, with a regular distribution and minimal FKBP5 expression. After 10 weeks, GR expression became diffuse and predominantly cytoplasmic; this was accompanied by substantial FKBP5 expression in the cytoplasm, with evident GR/FKBP5 colocalization. After 20 weeks, GR expression was diffuse and irregular, with almost no overlap with the nucleus (DAPI), while the number of FKBP5-positive cells increased.

### 2.4. Effects of Long-Term Chronic Stress on Systemic Inflammation in Rats

**Figure 4:**
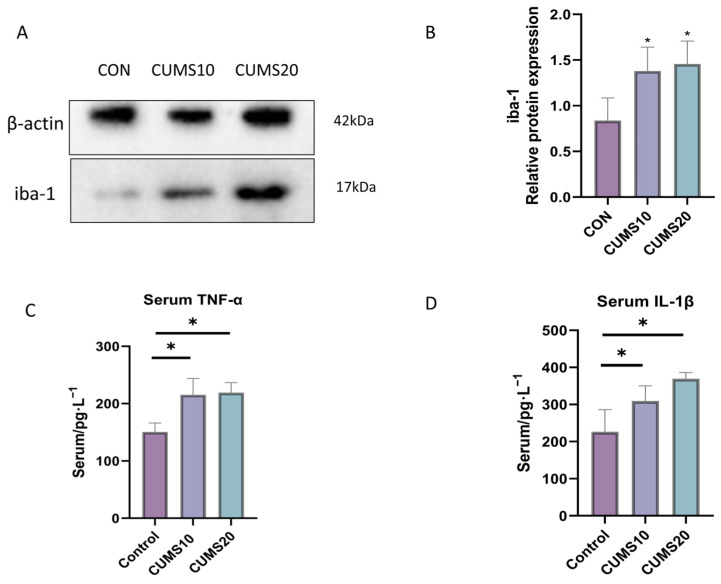
Activation of Inflammatory Markers in Rats After 10 Weeks of CUMS Exposure. (**A**,**B**) Western blot analysis of Iba-1 protein expression in the hippocampal tissue of rats across different groups (*n* = 3, *p* * < 0.05); (**C**,**D**) ELISA analysis of TNF-α and IL-1β levels in rat serum.

ELISA analysis of TNF-α and IL-1β levels in rats (Figure 4C,D) revealed a significant increase in inflammatory cytokines after 10 weeks of CUMS exposure (*p* < 0.05). However, no time-dependent increase in inflammatory cytokines was observed. Western blot analysis of hippocampal Iba-1 expression (Figure 4A,B) showed a significant increase in Iba-1 expression after both 10 and 20 weeks of CUMS exposure (*p* < 0.05), suggesting that chronic stress drives microglial activation and contributes to the inflammatory state.

### 2.5. Chronic Long-Term Stress Induces Bone Loss in Rats

**Figure 5:**
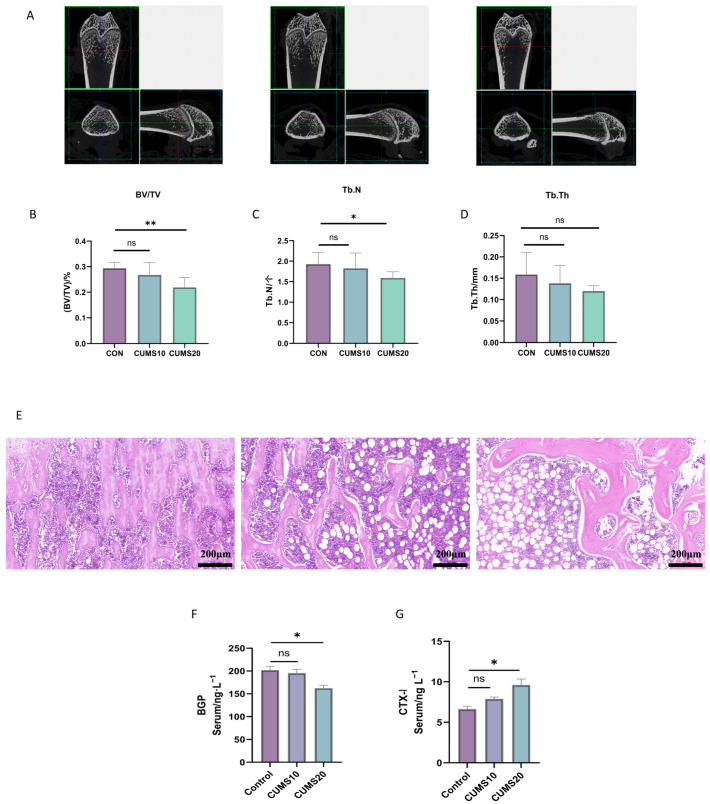

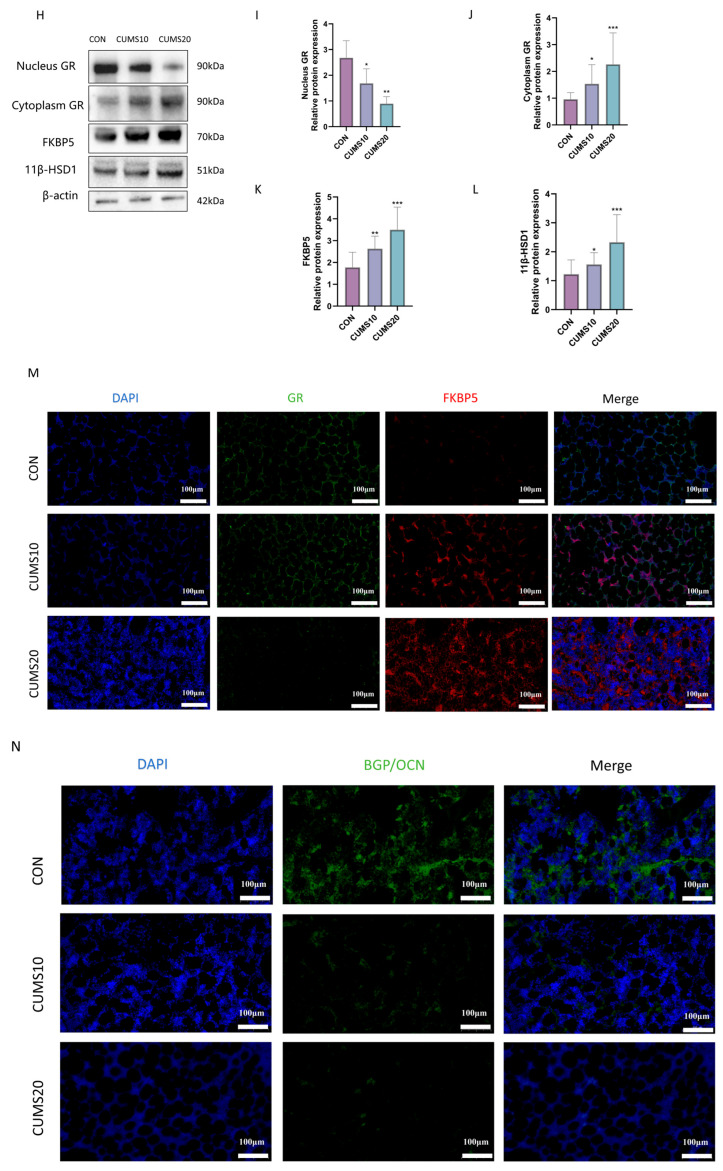

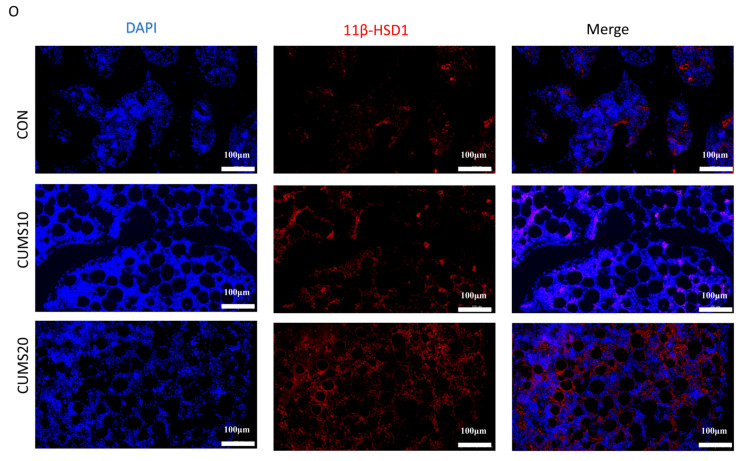
Reduced GR Nuclear Translocation in Osteoblasts Induces Bone Loss in Rats after Prolonged CUMS (Chronic Unpredictable Mild Stress) Exposure. (**A**) Micro-CT image of the right femur in rats, Representative coronal and cross-sectional Micro-CT images of the femoral neck; (**B**) Microstructural analysis of trabecular bone in the femur showing bone volume/tissue volume ratio (BV/TV) (*n* = 3, *p* ** < 0.01, ns > 0.05, ns: no significant difference); (**C**) Microstructural analysis of trabecular bone in the femur showing trabecular number (Tb.N) (*n* = 3, *p* * < 0.05, ns > 0.05, ns: no significant difference); (**D**) Microstructural analysis of trabecular bone in the femur showing trabecular thickness (Tb.Th) (*n* = 3, ns > 0.05, ns: no significant difference); (**E**) HE staining of rat femur (20× magnification), Nuclei are stained blue, while the cytoplasm and bone matrix are stained pink; (**F**) Relative levels of BGP (Osteocalcin) in the serum of rats across different groups (*n* = 5, *p* * < 0.05, ns > 0.05); (**G**) CTX-I (C-terminal telopeptide of type I collagen) in the serum of rats across different groups (*n* = 5, *p* * < 0.05, ns > 0.05); (**H**–**L**) Western blot analysis of nuclear GR, cytoplasmic GR, FKBP5, and 11β-HSD1 protein expression in hippocampal tissue across different groups (*n* = 3, *p* * < 0.05, *p* ** < 0.01, *p* *** < 0.001); (**M**) Immunofluorescence double staining of GR/FKBP5 in rat femur; (**N**) Immunofluorescence of BGP in rat femur; (**O**) Immunofluorescence of 11β-HSD1 in rat femur.

Micro-CT analysis (Figure 5A–D) showed that the trabecular bone volume and tissue volume ratio (BV/TV) were significantly reduced in the 20-week CUMS group compared to controls (*p* < 0.01), whereas no significant difference was observed at 10 weeks (*p* > 0.05). The number of trabeculae (Tb.N) in the CUMS 20-week group was significantly reduced (*p* < 0.05), while trabecular thickness decreased slightly, with no significant difference compared to the normal group (*p* > 0.05). Histopathological analysis (Figure 5E) revealed significant damage to the trabecular structure in the CUMS 20-week group, characterized by disrupted trabecular continuity, thinning, and fatty infiltration. The bone marrow cavity was notably enlarged, with increased fat cells and reduced hematopoietic cells. In contrast, the CUMS 10-week group displayed milder pathological damage, though trabecular fractures began to appear.

Serum ELISA (Figure 5F,G) indicated a decrease in BGP and an increase in CTX-1 in CUMS-exposed rats, reflecting suppressed osteoblastic activity and enhanced osteoclastic resorption. These metabolic alterations were duration-dependent; specifically, the 20-week group exhibited significantly lower BGP and higher CTX-1 levels compared to both the 10-week and control groups (*p* < 0.05). In contrast, BGP and CTX-1 levels in the 10-week group did not differ significantly from controls (*p* > 0.05).

To further investigate the expression of GR in the tibia, we examined the expression of nuclear GR, cytoplasmic GR, and associated chaperone molecules (Figure 5H–L). Results showed a significant reduction in GR nuclear translocation at 10 weeks of CUMS exposure (*p* < 0.05), which became more pronounced at 20 weeks. Conversely, expression of the chaperone FKBP5 increased significantly at 10 weeks (*p* < 0.05) and remained significantly elevated relative to controls at 20 weeks (*p* < 0.001). Local 11β-HSD1 expression exhibited a significant increase at 10 weeks (*p* < 0.05) and was markedly higher at 20 weeks (*p* < 0.001). Immunofluorescence (Figure 5M) analysis revealed a marked reduction in GR abundance near the trabeculae at 10 weeks. Furthermore, negligible nuclear colocalization of GR was observed at both 10 and 20 weeks, suggesting impaired glucocorticoid sensitivity. Immunofluorescence (Figure 5N,O) analysis demonstrated that BGP expression surrounding the trabeculae was prominent in the control group but showed a marked reduction at 10 weeks, Conversely, local 11β-HSD1, which was barely detectable in the control group, exhibited a significantly increased fluorescence signal beginning at week 10.

## 3. Discussion

### 3.1. Chronic Stress Dysregulates the HPA Axis and Impairs GR Nuclear Translocation

The hypothalamic–pituitary–adrenal (HPA) axis maintains physiological homeostasis via glucocorticoid-mediated negative feedback. A key finding of our study is that chronic stress exposure progressively disrupts this regulatory circuit, culminating in a state of profound HPA axis dysregulation. We demonstrate that prolonged stress impairs glucocorticoid receptor (GR) nuclear translocation in critical HPA-regulatory brain regions, including the hippocampus and prefrontal cortex. This dysfunction is characterized by a time-dependent decrease in nuclear GR abundance alongside increased cytoplasmic retention, effectively blunting the negative feedback signal required to terminate the stress response.

This impairment in GR trafficking is central to the observed HPA axis dysfunction. Reduced nuclear GR entry in hypothalamic and hippocampal neurons compromises their ability to suppress CRH and ACTH release, thereby perpetuating HPA axis overactivation. Immunofluorescence confirmed this subcellular pathology, revealing disorganized GR distribution and sequestration in the cytoplasm of neurons from stressed rats. This cytoplasmic retention of GR coincided with a marked upregulation of its inhibitory co-chaperone, FKBP5. Since FKBP5 sequesters GR in inactive cytoplasmic complexes and inhibits nuclear import, its overexpression provides a molecular mechanism linking chronic stress to impaired GR signaling and central glucocorticoid resistance.

Furthermore, the breakdown of GR-mediated signaling has profound implications for neuroinflammation. A primary function of nuclear GR is to transrepress pro-inflammatory gene expression. Our findings of increased microglial activation (elevated Iba-1) and increased cytokine levels (TNF-α, IL-1β) in the hippocampus of stressed rats are consistent with a failure of this crucial anti-inflammatory mechanism. This pro-inflammatory state is likely exacerbated by the significant overexpression of 11β-hydroxysteroid dehydrogenase type 1 (11β-HSD1) we observed in the prefrontal cortex and hippocampus. By locally regenerating active corticosterone from active glucocorticoids, amplified 11β-HSD1 activity creates a state of “local hypercortisolemia.” This sustained intracellular exposure can be neurotoxic and further desensitize GR, establishing a feed-forward loop of glucocorticoid resistance, inflammation, and neuronal damage [13,14,15], which ultimately drives the observed behavioral and physiological pathologies.

### 3.2. Impaired GR Nuclear Translocation Promotes Osteoblast Apoptosis

This systemic state of glucocorticoid resistance extends to skeletal tissue, acting as a primary driver of osteoblast apoptosis. The inhibition of glucocorticoid receptor (GR) nuclear translocation observed centrally is mirrored in peripheral osteoblasts, significantly compromising signaling efficiency consistent with resistance models [11]. The reduction in nuclear GR likely compromises the transcription of osteogenic genes (e.g., BGP) while disinhibiting pro-apoptotic pathways [16,17]. Importantly, we observed a distinct “local toxicity” phenomenon: despite the adrenal exhaustion and reduced serum CORT levels seen at 20 weeks, 11β-HSD1 was significantly upregulated within bone tissue. This osteotoxic effect is exacerbated by the observed pro-inflammatory milieu (elevated TNF-α, IL-1β), which accelerates bone resorption. together, these factors generate a toxic microenvironment that aggravates osteoblast apoptosis [18,19,20]. Immunofluorescence revealed GR and FKBP5 overexpression near osteoblasts, while histological analysis confirmed apoptosis, trabecular deterioration, marrow adiposity, and skewed remodeling, particularly at 20 weeks. Collectively, these findings substantiate the link between disrupted GR signaling and skeletal degeneration.

### 3.3. Chronic Stress Induces Time-Dependent Bone Loss

**Figure 6:**
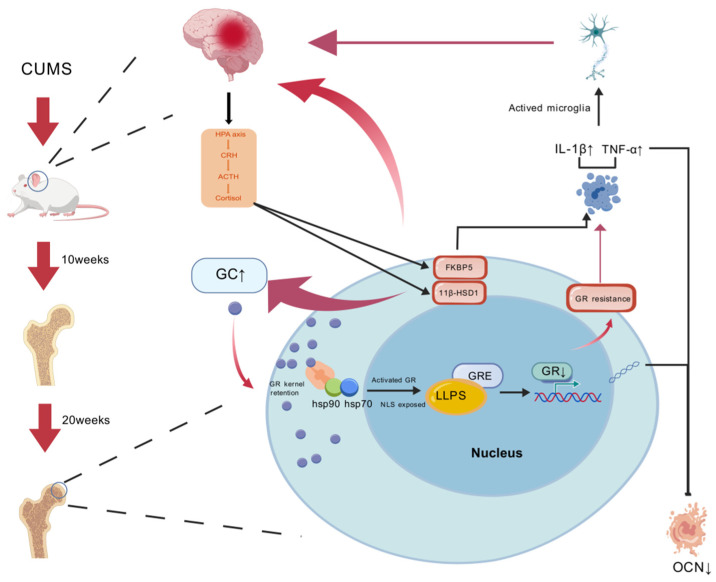
Chronic stress mediates bone loss through the HPA axis and GR nuclear translocation in a time-dependent manner. Exposure to CUMS in rat activates the hypothalamic–pituitary–adrenal (HPA) axis, resulting in increased secretion of corticotropin-releasing hormone (CRH), adrenocorticotropic hormone (ACTH), and glucocorticoids (GCs), such as cortisol. Elevated GC levels promote glucocorticoid receptor (GR) nuclear translocation and the activation of GR-responsive elements (GREs) via liquid–liquid phase separation (LLPS), where chaperone proteins hsp90 and hsp70 facilitate GR activation and nuclear localization. Meanwhile, sustained CUMS induces neuroinflammation via microglial activation, accompanied by increased expression of proinflammatory cytokines IL-1β and TNF-α, which contribute to GR resistance. Upregulation of FKBP5 and 11β-HSD1 further exacerbates this resistance. GR resistance impairs the transcription of downstream osteogenic genes such as osteocalcin (OCN), leading to decreased bone formation. Histological changes in bone are observed after 10 and 20 weeks of CUMS exposure, with significant reductions in trabecular bone mass at later stages. Arrows indicate the direction of regulation or influence; blunt-ended lines indicate inhibition. Picture created with BioGDP.com [21].

A central finding of this study is the time-dependent correlation between chronic stress duration and the severity of skeletal pathology (Figure 6). While bone abnormalities were detectable at 10 weeks, the extent of bone loss, microarchitectural deterioration, and cellular dysfunction was markedly more severe at 20 weeks. This temporal progression was mirrored in systemic biomarkers—notably the pronounced suppression of serum BGP—and in compromised trabecular microarchitecture, with the 20-week group exhibiting significantly lower bone mineral density than the 10-week cohort.

These data indicate that the pathogenic mechanisms identified—HPA axis dysregulation, central and peripheral GR dysfunction, FKBP5 upregulation, and local 11β-HSD1 amplification—cumulatively intensify with prolonged exposure. The progressive impairment of GR function shifts the remodeling equilibrium toward osteoblast apoptosis and uncoupled turnover, favoring resorption over formation. Clinically, these results underscore the importance of early intervention. Therapeutic strategies aimed at restoring HPA axis homeostasis, enhancing GR nuclear translocation (potentially by targeting FKBP5), or suppressing 11β-HSD1 activity represent promising avenues for mitigating stress-related osteoporosis, particularly before structural damage becomes irreversible.

### 3.4. Limitations and Future Directions

We acknowledge several limitations. First, the relationship between FKBP5 upregulation, GR dysfunction, and bone loss remains associative; future gain- or loss-of-function experiments (e.g., pharmacological inhibition or genetic knockdown) are required to definitively establish causality. Second, osteoblasts were identified via anatomical localization rather than specific markers (e.g., Osteocalcin, Runx2) or primary cell isolation. Future confirmation using specific double-labeling techniques is warranted to verify cell-specific apoptosis. Finally, osteoclast activity was assessed via systemic CTX-1 levels; subsequent studies incorporating TRAP staining and histomorphometry are needed to directly quantify local osteoclast numbers and dynamics.

## 4. Materials and Methods

### 4.1. Animals and Experimental Design

Thirty male specific pathogen-free (SPF) Sprague-Dawley rats (150 ± 20 g) were obtained from Beijing Weitong Lihua Laboratory Animal Technology Co., Ltd. (Beijing, China). Animals were maintained in a controlled environment (22 ± 1 °C, 50–60% humidity) with ad libitum access to standard chow and water. Following a one-week acclimatization period, rats were randomly allocated into three groups (*n* = 10/group): Control, 10-week CUMS, and 20-week CUMS. The CUMS protocol entailed daily exposure to a randomized sequence of varied mild stressors (e.g., altered housing, food/water deprivation, physical restraint) to prevent habituation. Control animals were housed under identical conditions but without stress exposure. All experimental procedures complied with ethical standards and were approved by the Institutional Animal Ethics Committee (Permit No. IBTCMCACMS21-2308-04).

### 4.2. Reagents and Instruments

Antibodies were purchased from Wuhan Sanying Biotechnology Co., Ltd. (Wuhan, China): CRH (Cat. No. 26848-1-AP), HSD11b1 (Cat. No. 10928-1-AP), GR (Cat. No. 66904-1-IG), FKBP5 (Cat. No. 67874-1-IG), BGP/OCN (Cat. No. 23418-1-AP), IBA-1 (Cat. No. 10904-1-AP), and Beta Actin (Cat. No. 66009-1-IG). Secondary antibodies included HRP-conjugated Goat Anti-Rabbit IgG (H + L) (Cat. No. SA00001-2) and HRP-conjugated Goat Anti-Mouse IgG (H + L) (Cat. No. SA00001-1). ELISA kits for Rat CRH, ACTH, and CORT were obtained from Beijing Meibiao Biotechnology Co., Ltd. (Beijing, China). A nuclear extraction kit (Cat. No. SN0020) was also used.

### 4.3. Chronic Stress Modeling and Grouping

Thirty male SD rats were singly housed and acclimatized for 1 week before random allocation into three groups: control group, 10-week Chronic Unpredictable Mild Stress (CUMS) group, and 20-week CUMS group. The control group was maintained under standard conditions without stress. CUMS groups underwent stressors including 12 h fasting, 12 h water deprivation, 24 h light/dark cycle reversal, cold swimming (4 °C, 5 min), heat exposure (45 °C, 5 min), foot shock (30 V, 5 s shocks at 5 s intervals for 120 s), tail clamping (1 min), sensory stimuli (white vinegar odor or continuous flashing light for 6 h), and environmental changes (damp bedding or inverted cages). Stressors were randomized, applied non-consecutively, and each used three times on average. Rats received at least one stressor daily for 28 or 56 days. Post-modeling, behavioral tests (sucrose preference, open field, elevated plus maze) were conducted. Successful CUMS modeling was defined by significant reductions in sucrose preference index and vertical/horizontal activity scores compared to controls.

### 4.4. Behavioral Experiments

#### 4.4.1. Sucrose Preference Test (SPT)

To assess sucrose preference, two water bottles were placed in each cage. Rats were initially acclimated to drinking plain water for 2 days, followed by a 3-day training period with both bottles containing 1% sucrose solution. Subsequently, one bottle was filled with 1% sucrose solution and the other with plain water, with their positions swapped every 12 h to ensure further adaptation. The SPT was conducted on the first day of Chronic Unpredictable Mild Stress (CUMS) modeling and the day prior to tissue collection. Before each formal test, rats were deprived of food and water for 12 h. At the test onset, each cage received 200 mL of plain water and 200 mL of 1% sucrose solution simultaneously. The test spanned 12 h, during which bottle positions were exchanged once to eliminate positional bias. After 12 h, the consumption of plain water and sucrose solution was measured and recorded.

#### 4.4.2. Open Field Test (OFT)

The open field apparatus consisted of a square arena with a 1 m × 1 m base and 50 cm high walls. The floor was divided into 16 (4 × 4) grids, with the central 4 (2 × 2) grids designated as the central area. Prior to testing, rats were transferred to a quiet, dark, and temperature-controlled room for 30 min of acclimation, followed by 5 min in a plastic box (45 cm × 30 cm × 15 cm). A camera positioned above the arena recorded their activity over a 5 min period. Key metrics included time spent in the central area (s), resting time (s), and the number of entries into the central area. After each test, the arena was cleaned with 75% ethanol to remove excreta and odors, preventing interference with subsequent trials.

#### 4.4.3. Elevated Plus Maze (EPM)

Rats were individually placed in the central area of an elevated plus maze (EPM), comprising two open arms and two closed arms (each 50 cm long and 20 cm wide), elevated 50 cm above a black floor. The intersection of the arms formed the central area, monitored by an overhead camera. Following a 5 s adaptation period, the rat’s movement trajectory, time spent in the open arms, and total distance traveled were recorded over 5 min. After each trial, the maze was cleaned with diluted ethanol across the open arms, closed arms, and central area, and aired out to eliminate residual odors before testing the next rat.

#### 4.4.4. Forced Swim Test (FST)

Twenty-four hours before the formal experiment, rats were placed in 25 °C water for 5 min to acclimate, with the water depth preventing their limbs from touching the container bottom. They were then removed, dried, and returned to their cages. The following day, under identical conditions, rats were reintroduced to the water, and their immobility time was recorded over a 5 min period.

### 4.5. Micro-CT Scanning and Imaging

Fresh right femurs were harvested from rats and fixed in 4% paraformaldehyde for 48 h. Following fixation, samples were rinsed under running water for 3 h and subjected to Micro-CT scanning. Microfocus computed tomography was employed to acquire planar and three-dimensional reconstructed images of the distal third of the femur. Key parameters calculated included tissue volume (TV), bone volume (BV), trabecular number (Tb.N), and trabecular thickness (Tb.Th).

### 4.6. Pathological Sections

#### 4.6.1. Hematoxylin–Eosin (HE) Staining for Morphological Analysis of Rat Hippocampus and Prefrontal Cortex

(1) Tissue Embedding and Sectioning: Fixed brain tissues were dehydrated through a graded ethanol series (75% for 4 h to anhydrous ethanol II for 30 min) and cleared in xylene (I and II, 15 min each). Tissues were then immersed in 65 °C paraffin three times (I, II, III, 1 h each) and embedded. After cooling at −20 °C, 5 μm thick sections were cut using a microtome and baked at 60 °C for 2 h.
(2) Hematoxylin–Eosin (HE) Staining: Sections were dewaxed in xylene (I and II, 15 min each) and rehydrated through a graded ethanol series (anhydrous I/II, 6 min each, to 75%, 6 min). Nuclei were stained with hematoxylin for 3 min, rinsed twice with pure water, differentiated with hydrochloric acid alcohol for 15 s, and rinsed thrice with pure water. Sections were blued in 1% ammonia water for 1 min and rinsed under running water for 3 min. After dehydration in 85% and 95% ethanol (5 min each), the cytoplasm was stained with eosin for 5 min, followed by a single water rinse.
(3) Dehydration and Mounting: Sections were dehydrated through a graded ethanol series (80% for 1 min to anhydrous III for 3 min), cleared in xylene (I and II, 3 min each), and mounted with neutral resin.
(4) Observation: Sections were examined under a light microscope, and images were captured. In HE staining, nuclei appear blue, and the cytoplasm appears red.

The morphology of the rat bed nucleus of the stria terminalis was also observed under a light microscope.

#### 4.6.2. Nissl Staining for Morphological Analysis of Rat Hippocampus and Prefrontal Cortex

(1) Toluidine Blue Staining: Dewaxed and rehydrated sections were immersed in toluidine blue solution and incubated at 56 °C for 1 h, followed by rinsing with deionized water.
(2) Differentiation: Nissl differentiation solution was applied for several seconds, with differentiation monitored microscopically until the background was nearly colorless, followed by rinsing under running water.
(3) Dehydration, Clearing, and Mounting: Sections were rapidly dehydrated in 85% ethanol (10 s), 95% ethanol (2 min), and anhydrous ethanol I and II (3 min each), cleared in xylene I and II (5 min each), and mounted with neutral resin.
(4) Observation: After the mounting medium dried, sections were examined under a microscope, and images were captured for analysis.

#### 4.6.3. Histomorphological Analysis of Bone Tissue

Left femurs were fixed in 4% paraformaldehyde for 24 h, decalcified in 10% ethylenediaminetetraacetic acid (EDTA) solution for 1 month, embedded in paraffin, sectioned, and stained with HE.

### 4.7. Immunofluorescence

Immunofluorescence staining was employed to examine the co-expression of GR/FKBP5 in the hippocampus, prefrontal cortex, and femur, as well as CRH/11β-HSD1 in the prefrontal cortex and 11β-HSD1/BGP in the femur. Sections were baked at 67 °C for 2 h, dewaxed in xylene I and II (10 min each), and rehydrated through a graded ethanol series (anhydrous I/II, 95% I/II, 75% I/II, 3 min each), followed by thorough PBS washing. Antigen retrieval involved sequential application of reagents A and B, each incubated at 37 °C for 30 min, with PBS washes between steps. Endogenous peroxidase was blocked with a blocking agent at room temperature in the dark for 10 min, followed by washing and blocking with 10% rabbit serum for 30 min at room temperature. For the first round of antibody incubation and TSA signal amplification, primary antibodies were applied and incubated overnight at 4 °C. After washing, HRP-conjugated secondary antibodies were incubated at room temperature for 50 min. Following thorough washing, TSA dye was applied and incubated in the dark for 10 min, followed by TBST washing (3 times, 3 min each). For the second round, sections underwent microwave-assisted antigen retrieval in buffer, cooled, and incubated with OCN-specific primary antibodies overnight at 4 °C. After washing, fluorescent secondary antibodies were applied and incubated in the dark for 50 min at room temperature, followed by PBS washing. Nuclei were counterstained with DAPI for 10 min in the dark, followed by washing. Autofluorescence was minimized with quenching agent B for 5 min and a 10 min rinse under running water. Sections were mounted with anti-fade medium, and fluorescence images were captured based on dye excitation wavelengths for subsequent analysis.

### 4.8. Western Blot

Western blot analysis was performed to detect protein expression of CRH, GR, FKBP5, and 11β-HSD1 in the hippocampus, hypothalamus, and tibia. For each rat, 50 mg of brain and bone tissue was lysed in 1 mL and 500 μL of RIPA buffer, respectively, supplemented with PMSF (100:1 ratio; 10 μL for brain, 5 μL for bone). Protein concentration was quantified using the BCA method, and 20 μg of protein per lane was separated via SDS-PAGE (5% stacking gel, 10% separating gel) at 80 V for 30 min (stacking) and 100 V for 90 min (separating). Proteins were transferred to a 0.4 μm PVDF membrane at 100 V for 120 min. Membranes were blocked with 5% skim milk in PBS for 2 h at room temperature on a shaker, washed with PBST (3 times, 5 min each), and incubated with primary antibodies (CRH 1:800, 11β-HSD1 1:400, GR 1:2000, FKBP5 1:3000, Iba-1 1:1000) overnight at 4 °C. After washing, secondary antibodies (goat anti-rat IgG 1:5000, goat anti-rabbit IgG 1:5000) were applied for 1 h at room temperature. Following additional washes, chemiluminescence was induced with ECL substrate, and images were captured using an automated imaging system. Grayscale values of protein bands were quantitatively analyzed. Data are expressed as mean ± SD. Western blot analyses were performed with *n* = 3 independent biological replicates per group, with each experiment repeated three times to ensure reproducibility.

### 4.9. Statistical Analysis

Data were analyzed using SPSS 26.0 and GraphPad Prism 10.0, with results expressed as mean ± standard deviation ($\overset{-}{x}$ ± s). Normality was assessed via the Kolmogorov–Smirnov test. For normally distributed data, one-way analysis of variance (ANOVA) was conducted to compare differences among the four groups, followed by Tukey’s post hoc test for multiple comparisons. A *p*-value < 0.05 was considered statistically significant.

## 5. Conclusions

The findings of this study demonstrate that prolonged chronic stress elevates serum cortisol concentrations, leading to structural atrophy in the hippocampus and prefrontal cortex. We show that sustained stress exposure progressively impairs GR function across the hippocampus, hypothalamus, and tibia. This impairment is characterized by reduced GR nuclear translocation and FKBP5-mediated interference with GC binding, which establishes a state of glucocorticoid resistance. Consequently, these changes disrupt HPA axis negative feedback and trigger inflammation activation. These findings identify the HPA-GR axis as a critical pathway linking chronic stress to osteoporosis and suggest that targeting the GR-FKBP5 axis or local 11β-HSD1 may represent a promising avenue for future therapeutic development.

## Data Availability

The data used during this study are available upon reasonable request from the corresponding authors.
